# Adsorption-based atmospheric water harvesting device for arid climates

**DOI:** 10.1038/s41467-018-03162-7

**Published:** 2018-03-22

**Authors:** Hyunho Kim, Sameer R. Rao, Eugene A. Kapustin, Lin Zhao, Sungwoo Yang, Omar M. Yaghi, Evelyn N. Wang

**Affiliations:** 1 Department of Mechanical Engineering, Massachusetts Institute of Technology, 77 Massachusetts Ave, Cambridge, MA 02139 USA; 2 Department of Chemistry, Kavli Energy NanoScience Institute, and Berkeley Global Science Institute, University of California–Berkeley, Berkeley, CA 94720 USA; 3 Materials Sciences Division, Lawrence Berkeley National Laboratory, Berkeley, CA 94720 USA; 4 King Abdulaziz City for Science and Technology (KACST), Riyadh, 11442 Saudi Arabia

## Abstract

Water scarcity is a particularly severe challenge in arid and desert climates. While a substantial amount of water is present in the form of vapour in the atmosphere, harvesting this water by state-of-the-art dewing technology can be extremely energy intensive and impractical, particularly when the relative humidity (RH) is low (i.e., below ~40% RH). In contrast, atmospheric water generators that utilise sorbents enable capture of vapour at low RH conditions and can be driven by the abundant source of solar-thermal energy with higher efficiency. Here, we demonstrate an air-cooled sorbent-based atmospheric water harvesting device using the metal−organic framework (MOF)-801 [Zr_6_O_4_(OH)_4_(fumarate)_6_] operating in an exceptionally arid climate (10–40% RH) and sub-zero dew points (Tempe, Arizona, USA) with a  thermal efficiency (solar input to water conversion) of ~14%. We predict that this device delivered over 0.25 L of water per kg of MOF for a single daily cycle.

## Abstract (web summary)

Harvesting water from the atmosphere is an important solution to water scarcity, but doing so in arid climates is highly challenging. Here, the authors develop a metal-organic framework-based water harvesting device that can deliver over 0.25 L of water per kg of adsorbent over a single cycle at relative humidities of 10–40% and at subzero dew points.

## Introduction

Enabling access to fresh potable water in desert and arid regions is a critical challenge and tightly coupled to social and economic development^1^. Water scarcity is difficult to address in areas that are landlocked and have limited infrastructure, such that mature water purification technologies, i.e., reverse osmosis and multi-stage flash, are challenging to implement. Atmospheric water generators (AWGs) can take advantage of solar energy via photovoltaics (refrigeration-based)^2,3^ or solar thermal (sorption-based)^4–6^ to harvest moisture from air. Typical AWGs utilise refrigeration to cool large volumes of air well below the dew point to condense water. The amount of energy consumed to harvest water from the air dramatically increases as the humidity or ambient temperature decreases. Desert and arid regions, unfortunately, have day-time relative humidities (RH; *P*_vap_/*P*_sat_, vapour pressure over saturation pressure) as low as ~10% with a vapour content of approximately 3 L of liquid water for every one million litres of air. For these conditions, the dew point can be sub-zero, requiring a large amount of energy to freeze and collect water out of air. Though the typical night-time RH can be as high as ~40%, the lower ambient temperature (~20 °C) prevents water harvesting with refrigeration-based AWGs. As a result, the practical implementation of refrigeration-based AWGs is infeasible^3,7^.

AWGs that take advantage of solar-thermal processes are a promising alternative to capture and deliver water in arid regions. In this approach, a sorbent is first saturated with water from air and subsequently heated to release and condense the water^4–6,8^. By selecting the desired sorbent characteristics (e.g., shape and step position of the isotherm, saturation capacity, and binding energy), solar-thermal-driven water harvesting is viable and efficient even in low RH conditions. Recently, we demonstrated that metal−organic frameworks (MOFs) are particularly attractive because they can capture more water and require lower regeneration temperatures^4^ for its release compared to conventional sorbents (e.g., zeolites/silica gels or liquid brines). In addition, their step-like isotherms suggest that a small change in temperature and/or RH can lead to a large change in uptake and water release. Our previous proof-of-concept device showed that with MOF-801^4^, water harvesting is possible (Cambridge, MA, USA, with ~65% RH), with a temperature differential of ~45 K between the MOF layer and condenser. While this demonstration as well as several prior studies^5,6,8^ have shown that the approach is viable, demonstration under representative conditions of desert/arid climates has not been achieved. Here, we experimentally demonstrate an air-cooled MOF-801-based water harvesting device operating in the arid climate of southwestern United States (Tempe, AZ, USA) with a day-time RH as low as 10% and under sub-zero dew points. In addition, we analysed the water from the adsorption−desorption/condensation process with MOF-801 and confirmed that the MOF compound is stable to water and the metal ions and organic linkers do not contaminate the produced water.

Cyclic water harvesting (i.e., multiple adsorption−desorption cycles a day) is challenging in extremely arid regions due to the low day-time humidity (~10% RH) that prevents water adsorption, i.e., the RH is lower than the adsorption step of MOF-801. Consequently, to achieve maximum water production for a single cycle, in this work, we implemented significant design improvements over our prior proof-of-concept^4^. First, we optimised and engineered the device to completely saturate during the night-time humidity swing (20–40% RH). Second, with an optical concentration of less than 2× and buoyancy-assisted vapour transport during condensation, the overall thermal efficiency (i.e., product of latent heat of water and mass of harvested water per unit input solar energy) was estimated to be ~14%. This corresponds to an increase of ~5× in comparison to operation without optical concentration and enabled complete regeneration with MOF-801.

Operation in such arid regions also opens an interesting avenue for increasing water harvesting output with passive radiative cooling by leveraging the typically clear sky. The clear night sky and low vapour content in the atmosphere enables dissipation of long-wavelength (infrared) thermal radiation from the device to the cold sky to cool it below its ambient temperature. By facing the device to the sky during adsorption, a ~3 K temperature drop was achieved, which corresponds to an increase in 5–7% RH experienced by the adsorbent. This passive cooling can lead to opportunities to utilise other adsorbents that have their adsorption steps located beyond the typical levels of RH in specific regions.

## Results

### Device design and operation

**Fig. 1:**
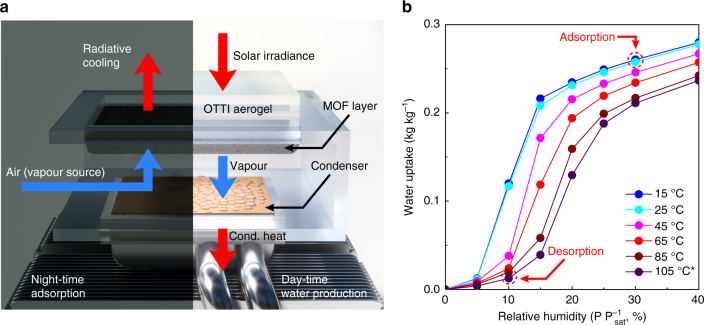
Working principle of the MOF-801-based water harvesting device and adsorption isotherms. **a** Illustrative schematic of the water harvesting device undergoing adsorption (night-time, left half) and solar-assisted water production (day-time, right half) processes. During adsorption, air is circulated around the MOF layer and water from air is adsorbed. Passive radiative cooling lowers the MOF layer temperature below the ambient by dissipating thermal radiation to the clear cold sky to increase the effective RH for adsorption. During water production, the OTTI aerogel is stacked on top of the MOF layer to suppress convective heat loss from the solar absorber. The desorbed vapour is condensed on a condenser and the heat of condensation is rejected to the ambient by a heat pipe heat sink. **b** Water adsorption isotherms of MOF-801 in kg kg^−1^ (kg of water per kg of MOF-801) as a function of relative humidity (P P_sat_^−1^, vapour pressure over saturation pressure) at temperatures of 15, 25, 45, 65 and 85 °C measured using a sorption analyser (Q5000 SA, TA Instruments). *The isotherm at 105 °C was predicted using the characteristic curve based on the isotherm at 85 °C^24^. The dotted red circles indicate representative conditions achieved during night-time adsorption and day-time water production in Arizona, USA

Our operational principle involves a single daily cycle where adsorption occurs during night-time at a higher humidity (20–40% RH) and solar-assisted desorption/water production occurs during day-time at a lower humidity (10–20% RH), schematically described in Fig. 1a. The device consists of two key components, an adsorbent layer (MOF) and an air-cooled condenser in an enclosure. The back side of the MOF layer is coated black and serves as a solar absorber. During night-time adsorption, the enclosure side walls are opened and the MOF layer is saturated with vapour from the natural flow of ambient air and passively cooled with radiation to the sky. During day-time water production, the enclosure is closed and the solar absorber side is covered with an optically transparent thermal insulator (OTTI aerogel)^9,10^. The MOF layer is heated by exposure to solar irradiance, causing water release (desorption). The desorbed water vapour diffuses from the MOF layer to the condenser due to a concentration gradient. Accumulation of vapour in the enclosure leads to saturation conditions and consequently, the condensation process occurs at ambient temperature. The heat of condensation is dissipated to the ambient by a heat sink. The adsorbents need to be selected based on the typically available ambient RH for water adsorption. MOF-801 was chosen in our study because it exhibits an adsorption step located around 20% RH and is well-suited for the specific climate tested (Tempe, AZ, USA). Furthermore, MOF-801 is hydrothermally stable and well-characterised for water adsorption including having high stability to cycling water in and out of the pores^4,11^.

The amount of water that can be harvested in a single cycle using MOF-801 can be evaluated based on the adsorption isotherm shown in Fig. 1b. For representative conditions in our test location, with a night-time ambient temperature of 15–25 °C and RH of ~30% during adsorption, the equilibrium uptake is estimated to be ~0.25 kg kg^−1^ (kg of water per kg of MOF-801). To achieve complete desorption (at ~10% RH, see Fig. 1b), with a day-time ambient (condenser) temperature of 30 °C (saturation vapour pressure, *P*_sat_ = 4.2 kPa), the adsorbent must be heated to a minimum of 77 °C (*P*_sat_ = 42 kPa). This corresponds to a target temperature difference of ~45 K between the adsorber and the condenser.

**Fig. 2:**
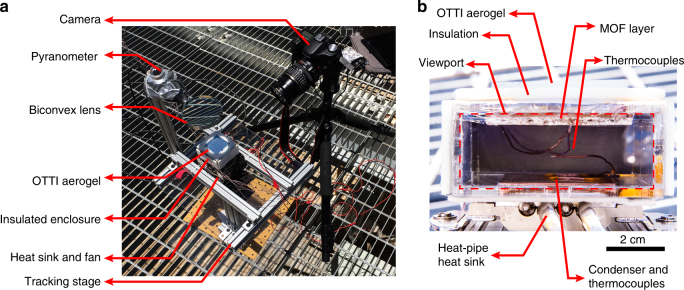
Water harvesting device test apparatus. **a** Photo of the device test apparatus during the solar-assisted water production with 1.8× optical concentration. Test location: Tempe, AZ, USA. **b** Photo of the water harvesting device showing the MOF layer (5 cm by 5 cm base, porosity of 0.67 or packing density of 464 kg m^−3^ with 2.57 mm thickness), condenser (4 cm by 4 cm), and thermocouples through the view port. The OTTI aerogel, heat pipe heat sink and insulation are also shown

To attain these operating conditions, based on the computational simulation (Supplementary Note 1), the prototype design described in our prior study was further optimised and engineered (Supplementary Note 4). A MOF layer (base of 5 cm by 5 cm with ~3 g of MOF-801) was fabricated using a porous copper foam. The solar absorber side of the MOF layer was coated with pyromark paint with a solar absorptance of ~0.95. The MOF layer density and thickness were optimised for operation in arid climates based on the transport properties of MOF-801. A packing porosity of 0.67 (or packing density of 464 kg m^−3^) and thickness of 2.57 mm were chosen for the MOF layer (see Supplementary Fig. 7). These optimised parameters enable saturation within the limited time window, i.e., during the humidity swing (increase) in the night-time (roughly under 8 h in a 20−40% RH environment) and to maximise water harvesting capacity. Due to the fixed side walls of the small-scale device (unlike Fig. 1a), which prevented access to air flow (vapour source), the MOF layer was secured in a separate enclosure that allowed adequate access to air (Supplementary Fig. 1). The condenser of the device was fabricated with a copper plate (4 cm by 4 cm and 0.6 cm thick) attached to a commercial air-cooled heat sink (NH-L9x65, Noctua) to efficiently dissipate the heat from condensation to the ambient. The condenser was air-cooled throughout the entire experiment. To suppress convective heat loss from the solar absorber side of the MOF layer during solar-assisted desorption, an OTTI aerogel with a thermal conductivity of less than 0.03 W m^−1^ K^−1^ and solar transmittance of ~0.94 (see Methods) was stacked on the MOF layer as shown in Figs. 1a,  2. The use of OTTI aerogel is well-suited for arid climates due to the inherently low RH and no degradation during testing was observed. In order to prevent vapour leak during desorption, a transparent plastic wrap (solar transmittance of ~0.93) was used to seal the device, leading to an overall solar transmittance and absorptance loss of ~17% (83% sun to thermal conversion efficiency) with an effective heat loss coefficient of 9−10 W m^−2^ K^−1^. To help overcome these solar-thermal losses and improve water harvesting thermal efficiencies, experiments were also performed with a biconvex lens (9 cm diameter) that was used to achieve an optical concentration of 1.8× during desorption. The spacing between the MOF layer and condenser (~1.8 cm) was also reduced in comparison to our prior study to enable faster vapour diffusion during condensation while maintaining minimal heat loss from the MOF layer to the condenser. One of the lateral walls of the device was made transparent to serve as a view port for visualisation.

### Water harvesting experiments

Five water harvesting cycles were performed between May 11 and May 18, 2017 (Tempe, AZ, USA) with the same MOF layer. Prior to the first cycle, the MOF layer was heated and dehydrated under direct solar radiation to ~50 °C and in an ambient of 35 °C and RH less than 20% for ~1.5 h. The experimental procedures and measurement/instrumentation details are presented in Methods. We initiated the water harvesting cycle around 20:00 hours local time. The absorber (black) side was positioned to face the clear sky to enable passive radiative cooling, reducing the MOF layer temperature below its ambient. Temperature drops of ~3 K were consistently observed throughout the adsorption phase of the five consecutive water harvesting cycles. This reduction in temperature corresponds to a 5−7% increase in effective RH experienced by the MOF layer. While the ambient vapour pressure was constant in this case, the saturation pressure is now defined by the temperature of the cooler adsorbent layer. After the overnight adsorption process, the MOF layer was installed back into the device between 06:00 and 07:00 hours local time, before the ambient RH started to decrease. The solar-assisted desorption phase of the water harvesting cycle started typically between 10:00 and 11:00 hours local time. For water harvesting cycles with non-concentrated solar irradiance, the global horizontal irradiance (GHI) was measured directly using a pyranometer. Water harvesting cycles with 1.8× optical concentration and direct normal irradiance (DNI) was achieved by facing the sun, where the measured global normal irradiance (GNI) was used to evaluate DNI for a clear day in Arizona, USA (see Methods). In addition, the tilting of the device at the elevation angle of solar irradiance enhanced mass transfer due to buoyancy-assisted transport and condensation of the hot desorbed vapour (see Supplementary Note 5). Due to the limited quantity of MOF-801 used in the device (~3 g), accurate measurement of the quantity of harvested water was not possible, albeit we expected ~0.75 g of water production. Therefore, we used validated computational predictions^4^ based on the measured conditions during the water harvesting cycles (ambient and condenser temperatures, RH, and solar flux) to evaluate the deliverable water capacity.

**Fig. 3:**
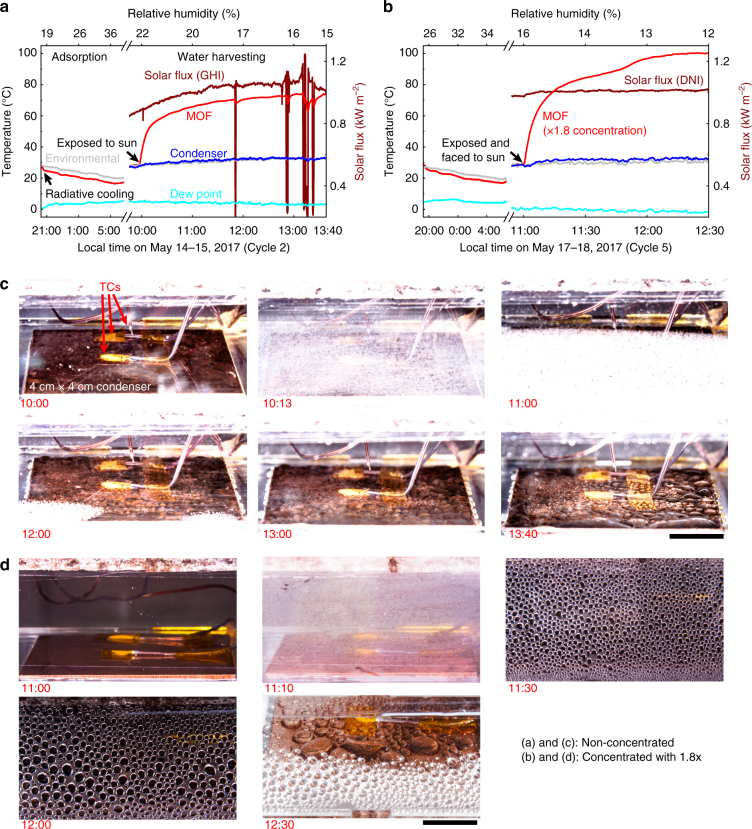
Water harvesting test results. **a**, **b** Representative temperature profiles (environmental, MOF layer, dew point and condenser) and solar flux (global horizontal irradiance (GHI) or direct normal irradiance (DNI)) as a function of local time for representative non-concentrated (Cycle 2, May 14−15, 2017) and concentrated with 1.8× (Cycle 5, May 17−18, 2017) cycles, respectively. **c**, **d** Representative photos illustrating droplet condensation on the copper plate condenser (4 cm by 4 cm) during the water harvesting process as a function of local time for representative non-concentrated (cycle 2) and concentrated (cycle 5) cycles, respectively. Shortly after the solar exposure, the view port fogged up due to condensation of desorbed vapour for both cycles. Thermocouples (TCs) measuring the condenser, air gap and the MOF layer temperatures are also shown. Due to the higher solar flux with the concentration, the rate of temperature increase of the MOF layer was significantly faster than the non-concentrated cycle, reducing the time required for desorption. The temperature slope change at ~11:45 hours local time indicates near completion of desorption. The predicted amount of harvested water for the non-concentrated (cycle 2) and concentrated (cycle 5) cycles were ~0.12 L and ~0.28 L per kg of MOF, respectively. Scale bars are 1 cm

Representative water harvesting cycles without optical concentration (May 14–15, 2017; see Supplementary movie 1) and with concentration (May 17–18, 2017) with the associated temperature profiles (MOF layer, environmental, dew point and condenser), solar flux and RH measurements are shown in Fig. 3a, b, respectively. In both figures, the upper abscissa indicates the measured RH at the local time of day (lower abscissa). The radiative cooling during the adsorption phase (between ~20:00 and 06:00 hours) is also shown. During the desorption phase (starting between ~10:00 and 11:00 hours the next day), the MOF layer temperature increased when exposed to incoming solar irradiation. Images taken during this phase are shown in Fig. 3c, d. The desorption started immediately following exposure to the solar irradiation and water condensation was observed on both the view port (fogging) and the condenser. The amount of fogging reduced over time as the enclosure walls and the air−vapour mixture inside the device heated up.

For the cycles carried out with optical concentration, the higher desorption temperatures (or desorption driving potential) can be inferred from Fig. 3b. In addition, the rate of regeneration was significantly faster than the predictions (see Supplementary Note 5 and Supplementary Fig. 10) due to buoyancy-assisted vapour transport during condensation (as the stage was tilted and faced the sun at the elevation and azimuth angles). The higher desorption temperatures associated with the concentrated case enabled complete desorption and this can be qualitatively deduced from the change in slope of the adsorber temperature in Fig. 3b (~11:45 hours local time). After complete desorption, most of the incident solar energy was available for sensible temperature rise leading to the slope change.

### Prediction of harvested water

High-fidelity computational simulations based on the characteristics of MOF-801 (Supplementary Figs. 9 and 10) were used to predict the water harvesting capacity of the device. Experimentally measured ambient and condenser temperatures, solar flux and RHs were used for the initial and boundary conditions. For the representative non-concentrated cycle, ~0.12 L of water per kg of MOF-801 was delivered following saturation at 40% RH (equilibrium uptake of ~0.28 kg kg^−1^). From the equilibrium considerations presented in Fig. 1b, after the desorption phase, the residual uptake at ~13% RH (adsorber at 74 °C and *P*_sat_ of 37 kPa; condenser at 33 °C and *P*_sat_ of 5 kPa) was ~0.09 kg kg^−1^, leading to a net water production capacity of ~0.19 L kg^−1^ (litres per kg of MOF). However, due to the kinetic limitations, the residual uptake at the end of the desorption was predicted to be only ~0.16 kg kg^−1^, leading to ~0.12 L kg^−1^ water production capacity. The kinetic limitations are dictated by intra/intercrystalline diffusion within the MOF layer as well as the vapour diffusion between the MOF layer and condenser. Similarly, for a representative cycle with an optical concentration (1.8×), an adsorber temperature of 100 °C (*P*_sat_ of 101 kPa) and a condenser temperature of 33 °C (*P*_sat_ of 5 kPa), the water production capacity was predicted to be ~0.28 L kg^−1^. This prediction is consistent with the estimate from the simulation (Supplementary Fig. 10). Here, the kinetic limitations were overcome by the higher adsorber temperature as well as the buoyancy-assisted vapour transport during condensation.

As a result of the optical concentration and tilting of the device, we predicted a thermal efficiency gain of ~5× in comparison to the non-concentrated cycle. Accordingly, with the concentration, the thermal efficiency was ~14% with input solar energy (product of DNI and optical concentration; herein, DNI was ~93% of GNI for the test location) and was ~3% for the non-concentrated cycle with GHI. The present device configuration and ambient conditions can deliver over ~0.34 L m^−2^ cycle^−1^ (litres per m^2^ of MOF layer base area per cycle) with the 1.8× solar concentration.

### Water quality analysis

Though hydrothermal stability of MOF-801 has been extensively studied and well-established^4^, we quantitatively characterised the quality of the harvested water using a bench-top adsorption cycling system that enables sufficient water collection (see Methods and Supplementary Fig. 11). Results from inductively coupled plasma-mass spectroscopy (ICP-MS) analysis indicate that the zirconium (metal ion of MOF-801) concentration in the water was found to be less than 1 ppb (parts per billion) as shown in Supplementary Fig. 12. In addition, the harvested water was analysed using infrared spectroscopy and evidence of organic linkers (fumarate) was not found, indicating that the compositions from MOF-801 did not contaminate the harvested water (see Supplementary Note 6 and Supplementary Fig. 13).

## Discussion

The concept of using night-time radiative cooling to increase the effective RH experienced by the adsorber layer was also introduced and discussed in this work. This approach opens possibilities to use MOFs with higher water uptake values and even lower regeneration temperatures, such as MOF-841^11^ or Co_2_Cl_2_BTDD^12^, in climates with ~20% RH even though their adsorption step is located at 25–30% RH.

FAM Z-series (functional adsorbent material zeolite), also referred to as AQSOA series^13^, offer step-like isotherms located at 15–25% RH with adsorption capacities of ~0.2 kg kg^−1^ (AQSOA Z01 and Z05). The device design and operation presented in this work can be similarly extended with such advanced zeolites. However, the synthetic flexibility and ability to tune adsorption properties are unique to MOFs^14,15^ and, therefore, ideally suited for atmospheric water harvesting systems. While the chosen MOF-801 in this study can deliver ~0.34 L m^−2^ cycle^−1^ (or ~0.28 L kg^−1^ cycle^−1^), further improvements can be realised with the development of new MOFs. For instance, with an identical device design and optimisation, a cobalt-based MOF (Co_2_Cl_2_BTDD)^12^ with an adsorption capacity greater than 0.8 kg kg^−1^ at ~30% RH can lead to ~1 L m^−2^  cycle^−1^ of water output, potentially surpassing the water harvesting output of advanced zeolites. While sorption kinetics of this MOF is relatively slower than MOF-801, we envision that the development of new MOFs with enhanced sorption capacities and kinetics can ultimately lead to a significant increase in water harvesting output.

Passive operation can be enabled with concentrating thermal energy with larger absorber areas^16^ or with stationary reflectors^17^ that eliminates the need for solar tracking (also see Supplementary Note 7). Furthermore, considerations presented in this work can be extended to a higher output system by integrating multi-layer adsorbent stacks into a compact bed-type architecture^18–20^, common to many classes of adsorption systems. The merit of the bed-type architecture is that in addition to solar-thermal, waste heat or low-infrastructure sources of energy such as biomass can be used to drive the desorption process (eliminating the need for a planar adsorber). While such a system configuration can enable higher output, the limitation of this approach is the need for auxiliary components (e.g., pumps) and higher system complexity to efficiently route the thermal energy to the various layers in the bed. The required heat storage capacity and source temperatures would need to be determined based on the required temperature difference between the adsorber and the condenser, which can be inferred from the adsorption isotherm. Our demonstration in an exceptionally arid climate indicates that adsorption-based water harvesting strategy is a promising solution to solve water scarcity in these regions.

## Methods

### Synthesis and characterisation of microcrystalline powder MOF-801

In a 500 mL screw-capped jar, 5.8 g (50 mmol) of fumaric acid (Fluka, 99%) and 16 g (50 mmol) of ZrOCl_2_·8H_2_O (Alfa Aesar, 98%) were dissolved in a mixed solvent of DMF and formic acid (200 and 70 mL, respectively). The mixture was then heated in an isothermal oven at 130 °C for 6 h to give as-prepared MOF-801 as white precipitate. The precipitate from three reaction jars was collected by filtration apparatus using a membrane filter (45 µm pore size), washed three times with 100 mL DMF, three times 100 mL methanol, and dried in air. Air-dried MOF sample was transferred to a vacuum chamber. The chamber was first evacuated at room temperature for 5 h until the pressure dropped below 1 kPa. After that, the sample was heated in vacuum at 70 °C for 12 h, and then at 150 °C for another 48 h. This finally gave activated MOF-801 as a white powder (yield: 30 g).

Low-pressure gas (N_2_ and Ar) adsorption isotherms (Supplementary Fig. 2) were measured using volumetric gas adsorption analyser (Autosorb-1, Quantachrome). Liquid nitrogen and argon baths were used for the measurements at 77 and 87 K, respectively. The powdered particle density (*ρ*_P_) of activated MOF-801 was estimated to be 1400 ± 20 kg m^−3^ from the pycnometer (Ultrapyc 1200e, Quantachrome) (skeletal density *ρ*_s_ = 2.6991 g cm^−3^) and BET pore volume measurements (*V*_P_ = 0.3425 cm^−3^ g) using the following equation: $\rho _{\mathrm{p}} = 1/(V_{\mathrm{p}} + 1/\rho _{\mathrm{s}})$. The particle size (see Supplementary Fig. 4) and intercrystalline diffusion characteristics of the powder MOF-801 were characterised and details are presented in Supplementary Note 2 and Supplementary Fig. 5. Additionally, the intra-crystalline diffusion characteristics were characterised as detailed in Supplementary Note 3 and Supplementary Fig. 6.

### Synthesis and optical characterisation of OTTI aerogel

The OTTI silica aerogel was synthesised by sol-gel polymerisation of tetramethyl orthosilicate (TMOS, 131903, Sigma Aldrich), using an ammonia solution (NH_3_, 2.0 M in methanol, 341428, Sigma Aldrich) as a catalyst to promote both hydrolysis and condensation reactions^9,10^. TMOS was diluted by methanol (MeOH, 322415, Sigma Aldrich) followed by addition of NH_3_ and water. The mixing molar ratio of chemicals was NH_3_:TMOS:water:methanol = 0.004:1:4:6. Then, the solution was gelled in a disposable polystyrene container. After 2 weeks, the container was dissolved away using acetone. The mother solvent was replaced with ethanol (EtOH, 89234-848, VWR) to be prepared for critical point drying (CPD, model 931, Tousimis) as EtOH is miscible with liquid CO_2_. To dry the wet gels in EtOH without cracks, it is important to dry them slowly to minimise capillary pressure during the CPD process. A bleed rate of 100 psi h^−1^ was used to decrease the CPD chamber pressure from ~1300 psi to ambient pressure. After drying, the monolithic aerogels were annealed at 400 °C for 24 h to maximise their transmittance. The aerogel was cut to the final size using a laser cutter (Epilog Zing). Experimentally measured solar transmittance and predicted thermal conductivity^21,22^ of the 8-mm-thick OTTI aerogel are shown in Supplementary Fig. 3a and b, respectively.

### Device fabrication

The adsorber layer was fabricated by first brazing a porous copper foam (~100 pores per inch or ppi), 0.26 cm thick, onto a copper plate (5 × 5 × 0.17 cm). The activated MOF-801 was infiltrated into this foam-plate structure by immersion drying in a ~50 wt. % aqueous dispersion. The copper foam provided structural rigidity and helped enhance the effective thermal conductivity of the layer, given the intrinsically low thermal conductivity of the porous MOF. The layer was then dried under vacuum for 4 h at temperature of 70 °C and the total mass dehydrated MOF-801 was characterised to be 2.98 g. This corresponds to a packing density of 464 kg m^−3^ (dry) and a porosity of 0.67. In order to enhance solar absorption, the back side of the adsorber was coated with Pyromark paint. This coating was optically characterised using a UV-Vis-NIR spectrophotometer (Cary 5000, Agilent) and found to have a solar-weighted absorptivity of 0.95.

The adsorber layer was then integrated into an enclosure constructed with acrylic sheets (0.318 cm thick). The top face was designed with a cut-out, equal in size to the adsorber layer (5 cm × 5 cm) and pilot holes to suspend the adsorber layer with nylon strings. Any gaps found between the side walls of the adsorber layer and the cut out were sealed with high temperature vacuum grease (Dow Corning). In addition, a layer of transparent polyethylene wrap was stretched over the entire top face and sealed against the side walls. Both these measures prevented leakage of any desorbed vapour. Thermal insulation (white in colour) was attached on all side walls except the view port. The adsorber side was completed by placing a piece of OTTI aerogel measuring 5 × 5 × 1 cm. The bottom face of the enclosure was made with a 4 × 4 cm cut-out to enable integration with a condenser assembly. The condenser assembly comprised a 4 × 4 × 0.6 cm polished copper piece that was bonded with high conductivity thermal epoxy (Omega Therm, Omega Engineering) to a heat pipe heat sink (NH-L9x65, Noctua). The air-cooled heat sink consisted of a finned heat pipe array with a fan that consumed ~0.9 W of electrical power to dissipate the condensation heat. The finished device measured 7 × 7 × 3.2 cm (excluding the heat sink, fan, insulation and aerogel) and was mounted on a stage with adjustable tilt to enable experiments under both GHI (no optical concentration, no tilt) and GNI (with optical concentration of 1.8× and tilt at elevation angles of 55−75° and azimuth angles of 100−180°).

### Experimental procedure

The water harvesting experiment comprises two phases: night-time vapour adsorption and day-time water harvesting and condensation. During vapour adsorption, typically started at 20:00 hours local time (UTC/GMT—7 h), the adsorber layer with its acrylic frame was mounted into the cover of an air-tight food storage container with the pyromark coated side up for night-time radiative cooling (Supplementary Fig. 1). The sides of the air-tight container were modified to fit a fan (0.9 W; 12 VDC) and enable cross flow of ambient air (vapour source). Two T-type thermocouple (5TC series, Omega Engineering) were used to measure the temperature of the adsorber layer during adsorption. In order to estimate the extent of radiative cooling and ambient temperature, another T-type thermocouple was placed in the air stream of another fan. Relative humidity measurements were made with a capacitive RH sensor (RH820U, Omega Engineering). Transparent polyethylene wrap was used to suppress convective heat loss on the black absorber side of the layer. Prior to the exposure to the clear sky, the container cover was wrapped in aluminium foil so that the MOF layer  could equilibrate with the ambient air.  Once the foil was removed, the MOF layer was exposed to the sky and an instant temperature drop of ~3 K below ambient was observed, as shown in Fig. 3a, b, due to radiative cooling. Adsorption was allowed to occur overnight and the sample was sealed into the device (between 06:00 and 07:00 hours local time) to prevent undesired loss of water due to the RH swing.

The procedure for water release and condensation typically started between 10:00 and 11:00 hours local time. In addition to the two T-type thermocouples embedded into the adsorption layer, three additional T-type thermocouples were used to measure temperatures: two for the copper condenser plate and one for the vapour space between the adsorber layer and the condenser plate. Ambient humidity and temperature conditions were recorded as described during the adsorption phase. The heat of condensation was dissipated to the ambient through the heat sink and fan operating at 0.9 W. The incoming solar irradiation (both global horizontal (GHI) and global normal (GNI) irradiations) was measured with a pyranometer (LP02-C, Hukseflux). The measured GNI was used to evaluate the DNI as: DNI = GNI – DI (diffuse irradiance) according to the weather data available from a weather station in Tucson, AZ, USA (available at NREL, SOLRMAP University of Arizona (OASIS)) for clear days in May 2017. The ratio between the GNI and DNI was found to be 0.93 and it matched well to an available correlation^23^. The area ratio between the lens and solar absorber surface was ~2.5; however, the achieved optical concentration of 1.8× was due to transmittance loss of the lens and the square solar absorber area being circumscribed by the circular concentrated solar irradiance (Fig. 2a). The actual optical concentration achieved with the concentrating lens was characterised to be 1.8× for the focal distance during the outdoor experiments with a thermopile detector (919P-040-50, Newport) and a solar simulator (92192, Newport Oriel). Solar transmittance of the transparent polyethylene wrap, ~0.93, was characterised with the pyranometer under direct solar irradiance. Images were acquired with a digital camera (EOS DS126211, Canon) to visualise the condensation process (see Fig. 3 and Supplementary movies 1 and 2). At the end of desorption, the MOF layer and its acrylic frame were extracted from the device to prevent re-adsorption of condensed water and isolated in an air-tight box. The adsorber assembly was only removed in the evening time to restart the adsorption phase for the next cycle. A total of five water harvesting cycles are reported in this study: cycle 1 (Supplementary Fig. 8a and b), cycle 2 (Fig. 3a, c), cycle 3 (Supplementary Fig. 8c and d and Supplementary movie 2), cycle 4 (Supplementary Fig. 8e and f), and cycle 5 (Fig. 3b, d).

### Water quality analysis

In order to quantitatively characterise the harvested water, a bench-top adsorption cycling system was constructed. A schematic of the water collection apparatus for ICP-MS analysis is shown in Supplementary Fig. 11a. The system consists of five main components, namely, adsorption and condenser chambers, a glass flask that serves as a reservoir for HPLC water (OmniSolv HPLC grade water, VWR), two temperature-controlled thermoelectric stages (CP-200HT-TT, TE Tech), and a vacuum pump. The adsorption and condenser chamber were custom-designed copper vacuum chambers (2 × 2 × 1 cm) with a removable lid. The adsorption chamber additionally had a layer of copper foam (2 × 2 × 0.8 cm) brazed to the bottom, which was infiltrated with activated MOF-801 (~1.5 g). These chambers were individually placed in thermal contact with a temperature-controlled thermoelectric stage that allowed for continuous cycling. Thermocouples (5TC series, Omega Engineering) were inserted into pilot holes made in the side walls of the copper chambers. For cycling, a pair of electronically controlled vacuum valves were used to link the adsorbent chamber to either the water reservoir during adsorption or the condenser chamber during desorption.

The adsorption−desorption cycles were performed under evacuated conditions to enable efficient transport of vapour across distances of ~0.5 m through the hoses and valves as shown in Supplementary Fig. 11a. The water in the glass flask was first degassed to remove non-condensable gases by connecting it to the vacuum pump and freezing the water. The flask was then heated to melt the ice under evacuation and reduce the solubility of non-condensable gasses. This cycle was repeated three times. The adsorption and condenser chambers were heated to 60 °C for 2 h under evacuated conditions to ensure there was no residual water in the system. The cycling experiments started with the adsorption phase, where the water reservoir was exposed to the adsorbent chamber. The dry adsorbent triggered evaporation and the generated vapour was adsorbed. During adsorption, the chamber was held at a constant temperature of 30 °C to extract the adsorption heat as well as prevent any condensation of vapour from the reservoir kept at ~20 °C. After complete adsorption (~40 min), the adsorption chamber was isolated from the water reservoir and exposed to the condenser chamber. The thermoelectric stage of the adsorption chamber was programmed to ramp up to 60 °C at this stage while the condenser stage was always maintained at 0.5 °C. The desorption was allowed to continue for 40 min at the end of which the adsorption chamber was opened to the reservoir and simultaneously cooled to 30 °C for the next cycle. Representative temperature and pressure profiles for a desorption−adsorption cycle are shown in Supplementary Fig. 11b. This cycle was repeated 18 times and about 8 g of condensed water was collected (i.e., ~0.3 L of water per kg of MOF per cycle).

The HPLC grade water from the reservoir was used as a control sample. The concentration of potentially contaminant elements was analysed using an inductively coupled plasma–mass spectroscopy system (ICP-MS, Agilent 7900, 68403A). Both the harvested water and control sample were analysed for the following elements: iron (from tubes/hoses), copper (from foam, chambers and braze alloy), silver, indium (both from braze alloy) and zirconium (from MOF compound). In addition, FT-IR spectra of control water (HPLC grade) and collected water from MOF-801 were collected in-house using a Bruker ALPHA Platinum ATR-FT-IR Spectrometer equipped with a single reflection diamond ATR module.

### Data availability

The authors declare that all data supporting the findings of this study are available within the article and its Supplementary Information or from the corresponding author upon reasonable request.

## Electronic supplementary material

Supplementary Information

Description of Additional Supplementary Files

Supplementary Movie 1

Supplementary Movie 2
