# Supplementary material for: Adsorption-based atmospheric water harvesting device for arid climates
Source: Nat Commun. 2018 Mar 22;9:1191. doi: 10.1038/s41467-018-03162-7 (PMC5864962; doi:10.1038/s41467-018-03162-7)
Supplement: Supplementary file 1 — Supplementary Information [file 41467_2018_3162_MOESM1_ESM.pdf]

Adsorption-based atmospheric water harvesting device for arid climates

Kim and Rao *et al*.

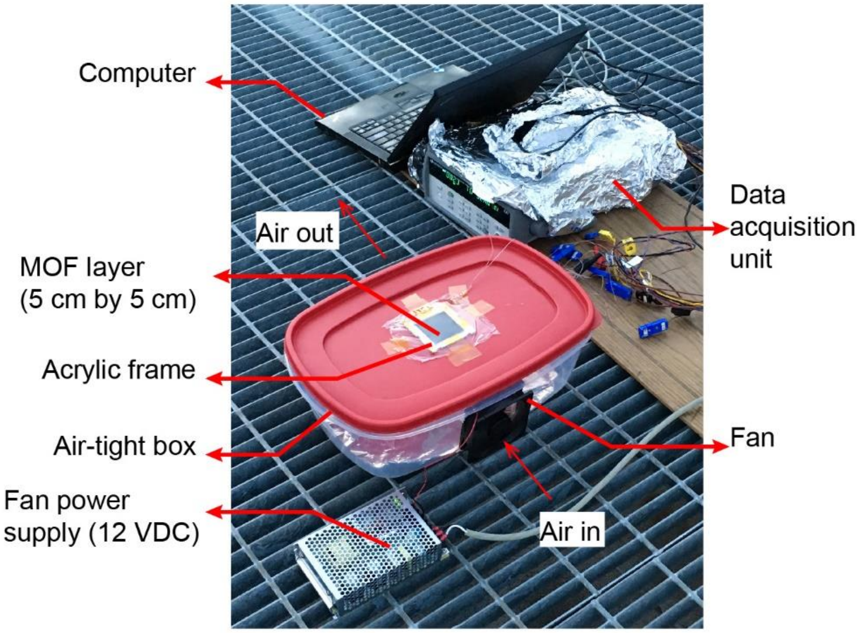

Supplementary Figure 1 – Photo of experimental setup during night-time adsorption. The MOF layer with its acrylic frame was mounted into cover of an air-tight plastic storage container with the pyromark coated side up for night-time radiative cooling. A ~5 kg metal block was placed inside to secure the apparatus against wind damage. The sides of the air-tight container were modified to fit a fan (0.9 W; 12 VDC) and enable cross flow of ambient air (vapour source). Initially, the black absorber side was covered with aluminium foil to reach thermal equilibrium with the ambient. At the start of the adsorption experiment, the aluminium foil was removed and a temperature drop due to the passive radiative cooling was observed (Figure 3 and Supplementary Figure 8).

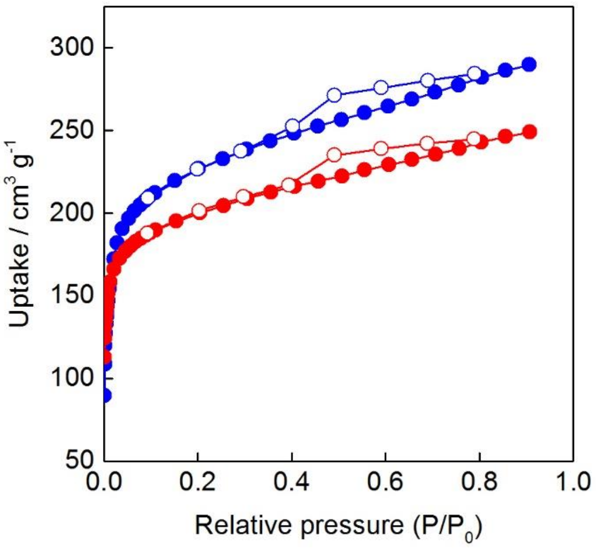

Supplementary Figure 2 – N_2 (red) and Ar (blue) adsorption isotherms of MOF-801 at 77 K and 87 K as functions of relative pressure, respectively.

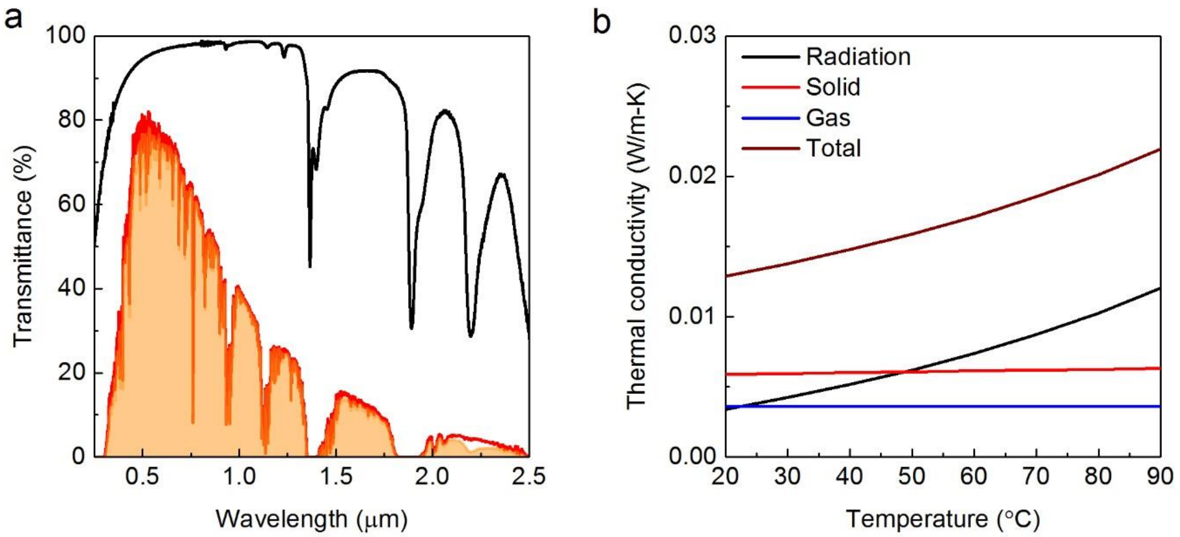

Supplementary Figure 3 – (**a**) Measured transmittance of an 8 mm thick aerogel sample using UV-Vis-NIR spectrophotometer (Cary 5000, Agilent). The AM1.5 solar spectrum is shown for comparison (red line). The orange area represents the transmitted spectrum by the aerogel. The solar weighted transmission of the sample is 94.5%. (**b**) Predicted thermal conductivity of an 8 mm thick aerogel sample. Contributions from radiation, solid conduction, and gas convection are also shown.

Supplementary Note 1: Theoretical modelling framework

A theoretical model based on mass and energy conservation was used to predict the adsorption-desorption dynamics and the extent of regeneration for MOF-801 during the solar-assisted desorption process using the following governing equations[1-3]:

$$\frac{\partial C}{\partial t} = \nabla \cdot D_{\mathrm{v}} \nabla C - \frac{(1-\varepsilon)}{\varepsilon} \frac{\partial C_{\mu}}{\partial t} \quad (1)$$

$$\rho c_{\mathrm{p}} \frac{\partial T}{\partial t} = \nabla \cdot k \nabla T + h_{\mathrm{ad}} (1-\varepsilon) \frac{\partial C_{\mu}}{\partial t} \quad (2)$$

Eqn (1) describes mass conservation of the vapour during diffusion and adsorption/desorption within the packed MOF layer. Here, C, is the local vapour concentration (mol m^{-3}), $\partial C_{\mu}/\partial t$ is the average instantaneous rate of vapour adsorption/desorption, ε is the porosity, and D_{v} is the intercrystalline diffusivity of vapour (m^2 s^{-1}). The vapour concentration (mol m^{-3}) can be expressed from the ideal gas law, $C = PR^{-1}T^{-1}$, where P, R, and T denote the pressure (Pa), universal gas constant (J mol^{-1} K^{-1}), and temperature (K). The effective vapour intercrystalline diffusivity, D_{v}, in an air-vapour mixture with consideration of both Knudsen and molecular diffusions in tortuous porous media can be estimated as[3-5],

$$D_{\mathrm{v}} = \varepsilon^{3/2} \left(\frac{1}{D_{\mathrm{vap}}} + \frac{1}{D_{\mathrm{K,vap}}} \right)^{-1} \quad (3)$$

where D_{vap} and $D_{\mathrm{K,vap}}$ are vapour molecular diffusivity in air and Knudsen diffusivity of vapour, respectively.

In Eqns (1) and (2), C_{μ} is the vapour concentration within an adsorbent crystal, and the average instantaneous rate of adsorption/desorption, $\partial C_{\mu}/\partial t$, can be approximated with the linear driving force model[1-3,6].

$$\frac{\partial C_{\mu}}{\partial t} = \frac{15}{r_{\mathrm{c}}^2} D_{\mu} (\mathrm{C}_{\mathrm{eq}} - C_{\mu}) \quad (4)$$

In Eqn (4), D_{μ}, represents the diffusivity of vapour inside an adsorbent crystal (intracrystalline), r_{c} is adsorbent crystal radius (m), and C_{eq} is the equilibrium vapour concentration corresponding to instantaneous local temperature and vapour pressure. r_{c} and D_{μ} were characterised experimentally, and C_{eq} can be estimated from a linear interpolation of the adsorption isotherms (Supplementary Figure 6 a).

Eqn (2) represents energy conservation within the MOF layer. Here, ρc_{P} represents the average heat capacity (J m^{-3} K^{-1}), k is the thermal conductivity (W m^{-1} K^{-1}), and h_{ad} is the enthalpy of adsorption (J mol^{-1}) for MOF-801 and water (~55 kJ mol^{-1}),[7]. The effective thermophysical properties were evaluated to include the contributions from the metallic copper foam (~3 W m^{-1} K^{-1}, porosity of ~0.95), MOF-801 (specific heat capacity of 760 J kg^{-1} K^{-1},[3]), and the adsorbed water (assumed to be in a liquid state). In Eqn (2), the advection term is neglected due to the high effective thermal conductivity of the MOF layer owing to the metallic binder (copper foam).

During the solar-assisted desorption, desorbed vapour is transported and condensed via diffusion in air. For the orientation of the device described in Figure 1 a, diffusional vapour

transport between the MOF layer and the condenser can be approximated using Fick's law of diffusion where x represents the spatial coordinate:

$$\frac{\partial C}{\partial t} = D_{\text{vap}} \frac{\partial^2 C}{\partial x^2} \tag{5}$$

Supplementary Note 2: Packing density and estimation of intercrystalline diffusivities

The effective intercrystalline diffusion is a function of spacing between the packed adsorbent particles and temperature. The characteristic void size of a random packing of spherical particles of uniform size can be estimated using a probability distribution[1,3,8], on the basis of its average packing porosity (ε) and the porosity corresponding to the maximum packing density of hexagonally packed spheres ($\varepsilon_{\mathrm{HCP}}$) as:

$$P(\chi) = 3(1+\chi)^2 \frac{\varepsilon(1-\varepsilon)(1-\varepsilon_{\mathrm{HCP}})}{(\varepsilon-\varepsilon_{\mathrm{HCP}})} \exp\left(-\frac{(1-\varepsilon)(1-\varepsilon_{\mathrm{HCP}})}{(\varepsilon-\varepsilon_{\mathrm{HCP}})} \left[(1+\chi)^3 - 1 \right] \right) \quad (6)$$

The average porosity (ε) was calculated from the measured MOF layer density (ρ_{layer}) and the estimated particle density (ρ_{p}) of the activated MOF-801 (1400± 20 kg m^{-3}) using Eqn (7). The porosity of the MOF-801 layer is determined to be 0.67.

$$\varepsilon = 1 - \frac{\rho_{\mathrm{layer}}}{\rho_{\mathrm{p}}} \quad (7)$$

The characteristic void size (d_{p}) based on this distribution can be estimated as, $d_p = 2r_{\mathrm{c}}X_{\mathrm{avg}}$, where r_{c} is the MOF crystal radius, and X_{avg} is defined as:

$$\chi_{avg} = \frac{1}{\varepsilon}\int_0^{\infty} \chi P(\chi) d\chi \quad (8)$$

The average crystal diameter of MOF-801 (~1 μm) was characterised using a scanning electron microscope (6010LA SEM, JEOL), as shown in Supplementary Figure 4, and assumed uniform for the estimation of the void size.

The effective intercrystalline diffusivity can be computed using Eqn (3), where the Knudsen diffusivity is $D_{\mathrm{K,vap}} = (d_{\mathrm{p}}/3)\sqrt{8RT/\pi M}$, and the d_{p} is ~0.34 μm for the porosity of 0.67 and uniform crystal diameter of 1 μm. The diffusion coefficient of vapour in air at atmospheric pressure as a function of temperature can be obtained using the following relation[4],

$$D_{\mathrm{vap,T}} = D_{\mathrm{vap,ref}} \left(\frac{T}{T_{\mathrm{ref}}} \right)^{3/2} \left(\frac{\Omega_{\mathrm{D,ref}}}{\Omega_{\mathrm{D,T}}} \right) \quad (9)$$

where D_{vap} and Ω_{D} are the vapour diffusion coefficient and collision integral, respectively, and subscript ref denotes reference value. Effective intercrystalline and vapour diffusivities in air are plotted in Supplementary Figure 5 using Eqns (3) and (9), respectively.

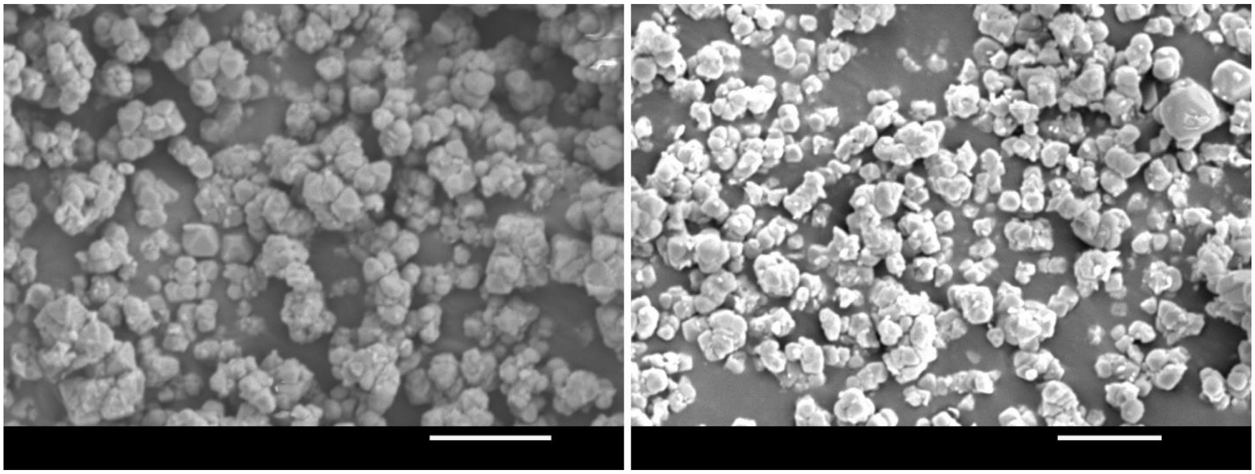

Supplementary Figure 4 – SEM images of powdered MOF-801. Crystal diameter of MOF-801 is 1 ± 0.15 μm, mean value and error (standard deviation) were obtained from image analysis using ImageJ software. Scale bars are 5 μm.

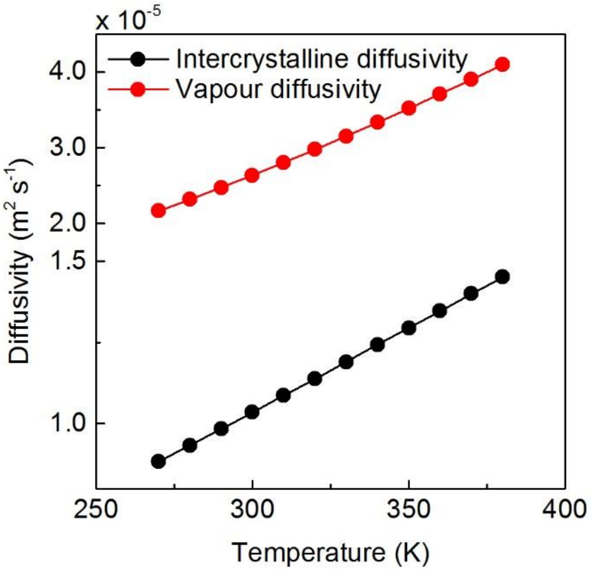

Supplementary Figure 5 – Effective intercrystalline vapour diffusivity of packed MOF-801 in air (black) as a function of temperature for the porosity of 0.67 and crystal diameter of 1 μm. Vapour diffusivity in air (red) as a function of temperature is also shown for comparison.

Supplementary Note 3: Adsorption isotherms and estimation of intracrystalline diffusivities of MOF-801

Vapour adsorption isotherms of MOF-801 were characterised using an adsorption analyser (Q5000 SA, TA instruments) at 15, 25, 45, 65, and 85°C (Supplementary Figure 6 a). The adsorption isotherm at 105°C was predicted using the characteristic curve based on the 85°C isotherm, vapour uptake as a function of adsorption potential: $A = RT\ln(P_{\mathrm{sat}}/P)$.[9] The vapour adsorption isotherm of MOF-801 before and after water harvesting cycles is also shown in Supplementary Figure 6 b, indicating the hydrothermal stability of MOF-801.

Using the dynamic adsorption behaviour (*i.e.*, rate of mass adsorbed as a function of time), intracrystalline vapour diffusivity of MOF-801 was estimated using the following relation[10] where we assume homogeneous pore structure, constant spherical adsorbent crystals of radius (r_{c}), and constant surface concentration and diffusivity (D_{μ}),

$$\frac{m_t}{m_{\mathrm{eq}}} = 1 - \frac{6}{\pi^2}\sum_{n=1}^{\infty}\left(\frac{1}{n^2}\right)\exp\left(-\frac{n^2\pi^2 D_{\mu} t}{r_{\mathrm{c}}^{2}}\right) \tag{10}$$

where m_t/m_{eq} is the fractional water uptake with $m_t = 0$ at $t = 0$ and $m_t = m_{\mathrm{eq}}$ as $t \longrightarrow \infty$ for a sufficiently small pressure and uptake step. The effective intracrystalline (Fickian) diffusivity of MOF-801 at 25 and 65°C were estimated by fitting Eqn (10) with the experimental measurements, as shown Supplementary Figure 6 c and d, respectively. For the macroscopic modelling framework outlined, it is essential to define a characteristic intracrystalline diffusivity[3], therefore, constant intracrystalline diffusivities at 25°C or 65°C (25% RH) were used for the theoretical prediction.

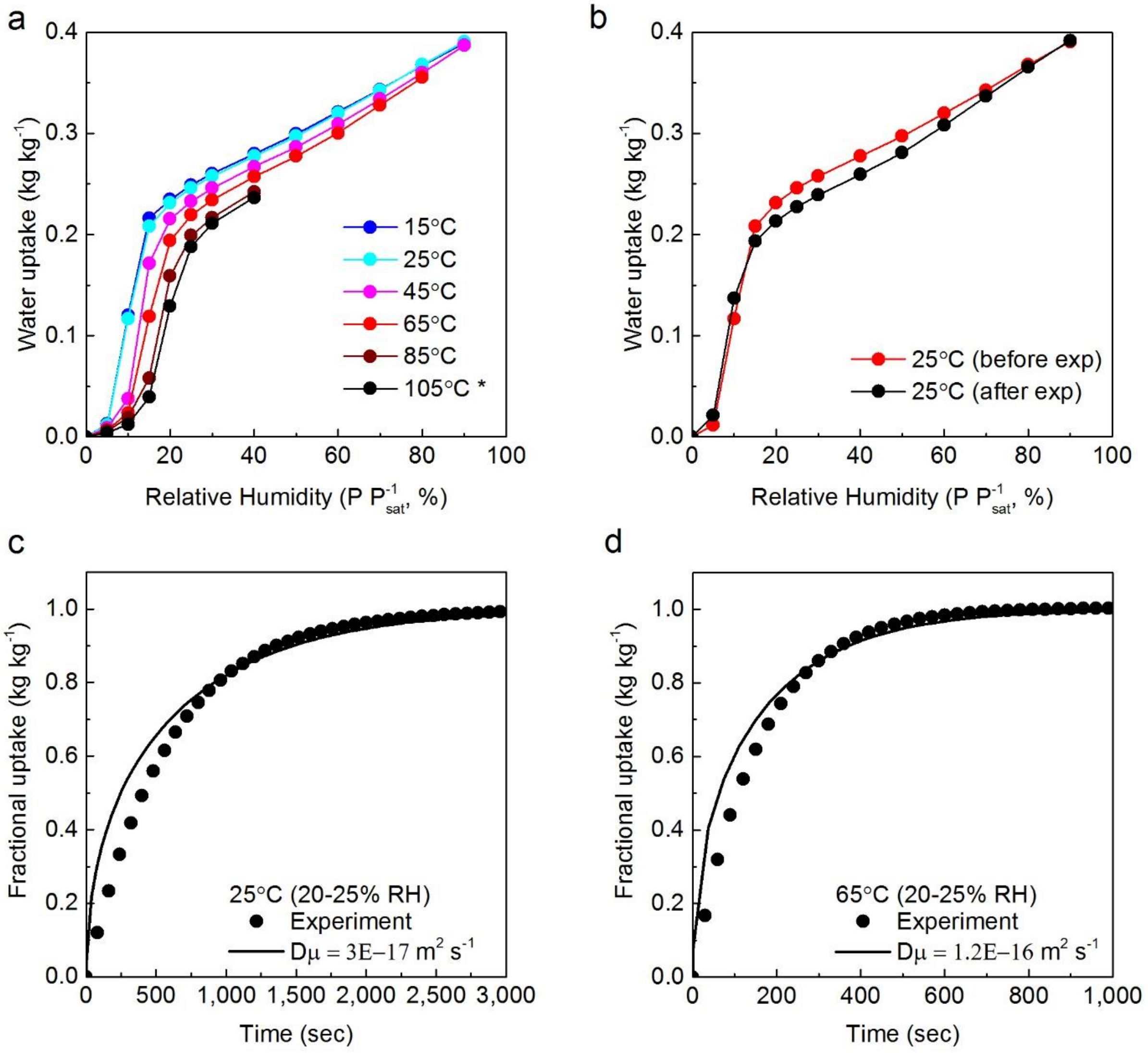

Supplementary Figure 6 – (**a**) Vapour adsorption isotherms of MOF-801 at 15, 25, 45, 65, and 85°C. *Isotherm at 105°C was predicted from the characteristic curve[9] based on the 85°C isotherm. (**b**) Vapour adsorption isotherms of MOF-801 at 25°C before (red) and after climate testing (black). (**c**) and (**d**) Fractional water uptake (kg kg^{-1}) as a function of time for MOF-801 characterised at 25 and 65°C at 25% RH, respectively. Dotted data (experimental) and solid line (fitting from Eqn (10)).

Supplementary Note 4: Optimisation of MOF layer

To enable sufficient vapour diffusion kinetics along with reasonably high water production, findings from our recent study[3] suggests that the optimum packing porosity for the MOF-801 based water harvesting device is ~0.7. Using the theoretical framework presented and characterised properties, adsorption-desorption dynamics for MOF-801 were simulated, as shown in Supplementary Figure 7, and used as a guideline for selecting the optimum MOF layer thickness.

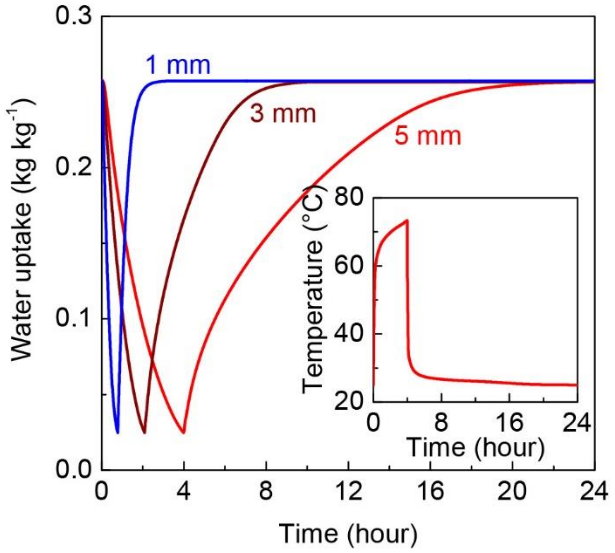

Supplementary Figure 7 – Adsorption-desorption dynamics of MOF-801 in ambient air at 30% RH. Predicted adsorption-desorption dynamics with a packing porosity of 0.67, desorption heat flux of 1 kW m^{-2}, natural convective heat transfer coefficient of 10 W m^{-2} K^{-1}, ambient temperature of 25°C, and thicknesses of 1, 3, and 5 mm. MOF-801 is initially equilibrated at 30% RH (25°C), and the partial vapour pressure rapidly increased from 30% RH to 100% RH (at 25°C) for condensation/water harvesting at 25°C with a desorption heat flux of 1 kW m^{-2}. Durations of solar exposure for 1, 3, and 5 mm thick MOF layer are 0.8, 2.1, and 4 hours, respectively. After desorption, solar exposure is stopped and the surrounding RH reverted to 30% RH for water adsorption from air. The temperature profile of a 5 mm thick MOF layer is also shown. Based on the predicted performance for the porosity of 0.67, the MOF layer thickness should be ~3 mm to enable complete saturation under the limited time window for adsorption (approximately under 8 hours in 20-40% RH environment). For simplicity, constant intracrystalline diffusion coefficient of 3E-17 m^2 s^{-1} is used for the simulation (Supplementary Figure 6 c) and sufficiently fast air freestream velocity is assumed to keep the RH of 30% at the MOF layer.

Supplementary Note 5: Water harvesting cycles – experiments and predictions

Using the theoretical framework outlined and characterised properties, computational simulations were carried out using COMSOL Multiphysics to evaluate the extent of regeneration during the solar-assisted water harvesting (Supplementary Figure 9 and Supplementary Figure 10). Temperature (MOF layer, environmental, dew point, and condenser) and solar flux profiles, and photos of condensed droplets of the water harvesting (adsorption-desorption) cycles for the cycle numbers 1, 3, and 4 are shown in Supplementary Figure 8. Note that cycles 1 to 3 were carried out under global horizontal irradiance (GHI), and cycles 4 and 5 were carried out with 1.8x optical concentration with direct normal irradiance (DNI). For the cycle 4 and 5, due to buoyancy-assisted condensation with tilting of the stage, the regeneration was significantly faster than the predictions as evident in the change in temperature slope shown in Supplementary Figure 10 after ~45 minutes of desorption. The thermal efficiency of the water harvesting cycle is defined as

$$\eta_{\mathrm{thermal}} = \frac{m_{\mathrm{water}} h_{\mathrm{fg}}}{Q_{\mathrm{solar}}} \tag{11}$$

where m_{water}, h_{fg}, and Q_{solar} are predicted amount of harvested water, latent heat, and input solar energy, respectively. For the non-concentrated cycle number 2, η_{thermal} is predicted to be ~3% (with GHI) and for the concentrated cycle number 5, η_{thermal} is predicted to be ~14% (with GNI times optical concentration of 1.8x). The efficiency for the concentrated cycle was evaluated on the basis of the time at which a change in the slope of the MOF temperature was observed. Despite the near complete desorption, at the time of the slope change, the simulation predicts ~0.1 kg kg^{-1} of residual uptake (Supplementary Figure 10). This is due to fact that the simulation does not take into account the enhanced vapour transport due to buoyancy. Furthermore, the enhanced rate of desorption driven by the enhanced vapour transport (lower interface vapour pressure) is evident from the lower MOF layer temperature observed in comparison to the simulations. This can also be qualitatively deduced from the significantly greater amount of water condensation on the viewport compared to the non-concentrated cycles.

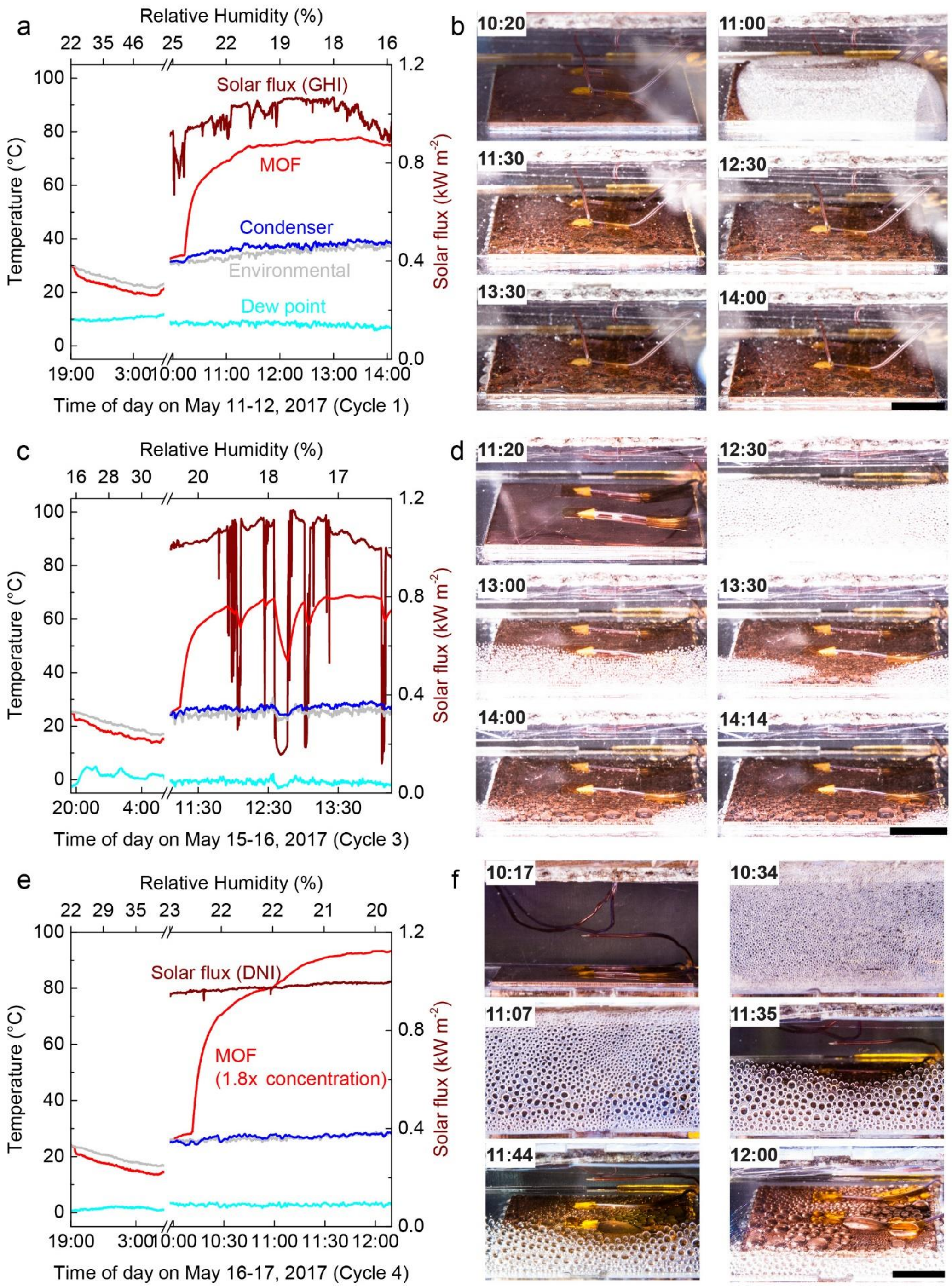

Supplementary Figure 8 – (**a**) Representative temperature profiles (environmental, MOF layer, dew point, and condenser) and solar flux (global horizontal irradiance (GHI)) as a function of local time for the cycle 1. (**b**) Representative photos illustrating droplet condensation on the copper condenser (4 cm by 4 cm) during desorption process as a function of local time for the cycle 1. (**c**) and (**d**), and (**e**) and (**f**) represent temperature profiles, solar flux (GHI for cycle 3 and direct normal irradiance (DNI) for cycle 4), and representative photos of droplet condensation for cycle number 3 and 4, respectively. Cycle 4 was carried out under DNI with optical concentration of 1.8x. Scale bars are 1 cm.

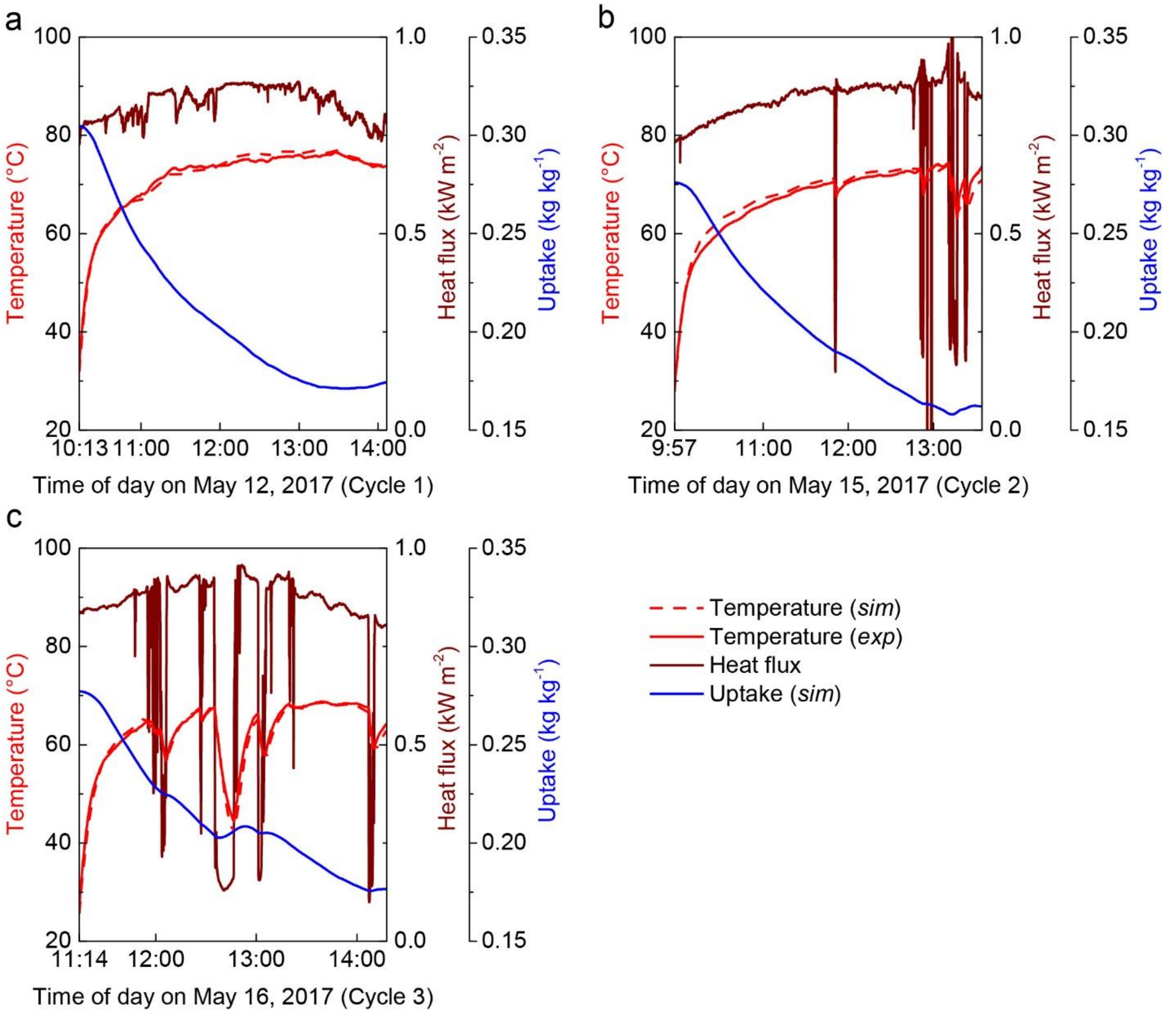

Supplementary Figure 9 – (**a**)-(**c**) Temperature profiles [Experimental (red solid line); predicted (red dotted line)], heat flux for desorption [(solar flux)*(optical and absorptance loss)], and predicted vapour uptake during the water harvesting as a function of time of day for cycles 1, 2, and 3, respectively. For cycle 1, an initial equilibrium RH of 55%, and for cycles 2 and 3, an initial RH of 40% was assumed based on the RH and radiative cooling measurements shown in Figure 3 and Supplementary Figure 8. For simplicity, a constant intracrystalline diffusion coefficient of 1.2E-16 m^2 s^{-1} was used for the simulation (Supplementary Figure 6 d).

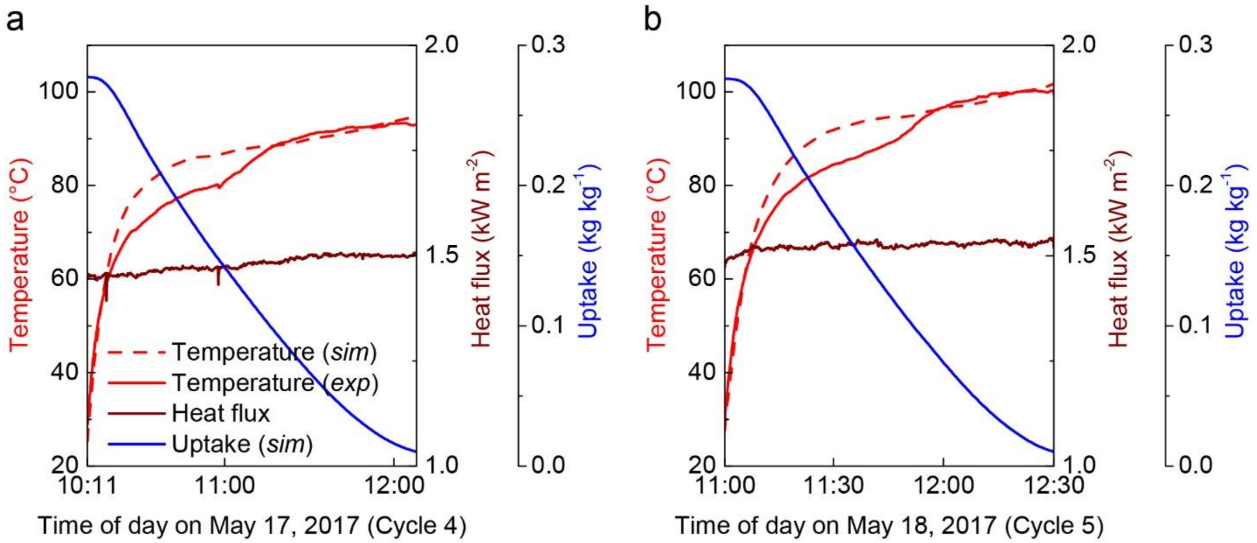

Supplementary Figure 10 – (**a**)-(**b**) Temperature profiles [Experimental (red solid line); predicted (red dotted line)], heat flux for desorption [(solar flux)*(optical and absorptance loss)*(optical concentration)], and predicted vapour uptake during water harvesting as a function of time of day for cycles 4 and 5, respectively. For both cycles, an initial equilibrium RH of 40% was assumed based on the RH and radiative cooling measurements shown in Figure 3 and Supplementary Figure 8. For simplicity, constant intracrystalline diffusion coefficient of 1.2E-16 m^2 s^{-1} was used for the simulation (Supplementary Figure 6 d).

Supplementary Note 6: Water quality analysis

Results shown in Supplementary Figure 12 indicate that zirconium concentration in both the collected and control water was found to be indistinguishable and less than 1 ppb (part per billion), indicating that the metal ions (Zr) from MOF-801 did not leach the harvested water. The largest difference in composition was found in concentrations of iron and copper (which are both absent in MOF-801 compound) due to oxidation reactions occurring during the cycling experiments. While the concentration of iron (~3 ppb) in the harvested water was negligible, the concentration of copper (~2.6 ppm) can be eliminated through material choices. Copper was chosen in this study for its high thermal conductivity and ease of machinability (*i.e.*, milling and fabrication of chambers), which enabled an isothermal condenser. In a practical system, we envision the use of galvanized steel as a candidate material for the condenser and thermal binder. The FT-IR spectra indicated that signature of organic linkers (fumaric acid) was absent.

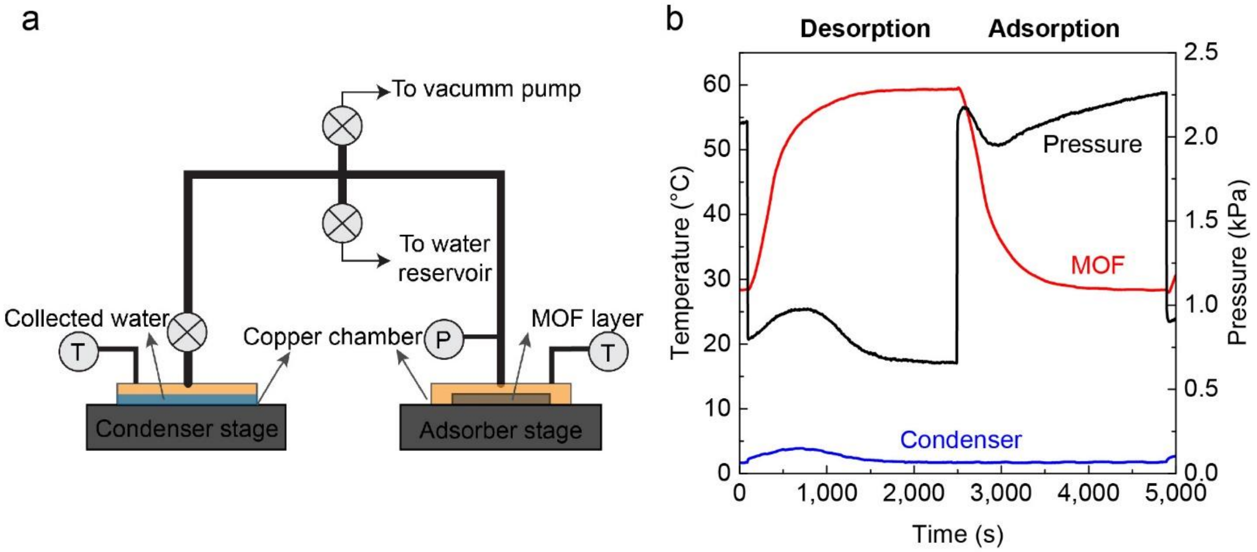

Supplementary Figure 11 – (**a**) Schematic of water collection apparatus with MOF-801 layer. (**b**) Representative temperature (MOF chamber and condenser chamber) and pressure profile (MOF chamber) for a desorption-adsorption cycle as a function of time.

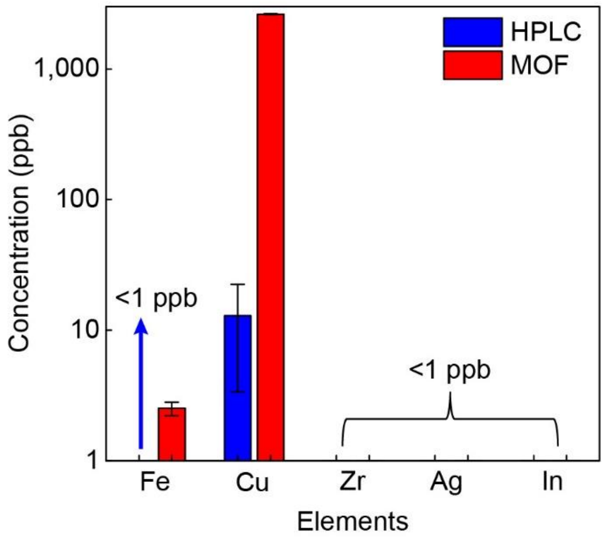

Supplementary Figure 12 – ICP-MS analysis of control water (HPLC) and water collected from MOF-801 (MOF). Iron (Fe; 56), copper (Cu; 63), zirconium (Zr; 90 and 91), silver (Ag; 107), and indium (In; 115) concentrations were analysed. Zirconium, silver, and indium concentrations in both HPLC and MOF samples were found to be less than 1 ppb (part per billion), indicating that the compositions from MOF-801 did not contaminate the harvested water. Iron concentrations in the harvested water (MOF) and control water (HPLC) were ~3 ppb and less than 1 ppb, respectively.

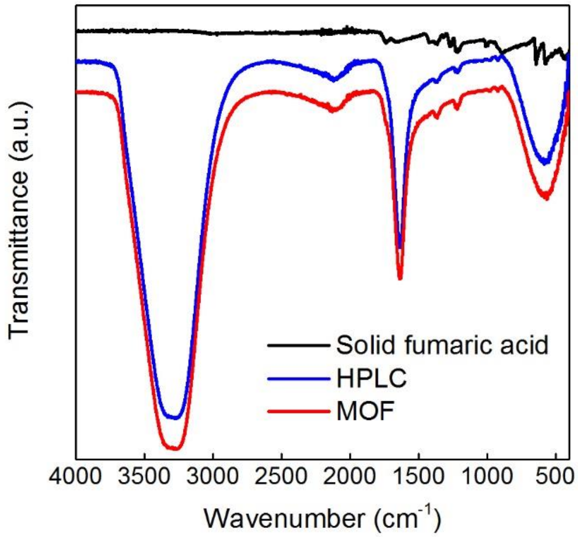

Supplementary Figure 13 – Comparison of FT-IR spectra (transmittance as a function of wavenumber) for solid fumaric acid, HPLC grade water (HPLC), and water collected from MOF-801 (MOF).

Supplementary Note 7: Thermal analysis for passive operation

Our proposed approach can harvest water solely based on solar-thermal energy without any additional input of electrical energy (*i.e.*, in a passive manner) for remote/arid climates. In this section, we present thermal analysis which shows that complete passive solar-thermal operation is realistic without any additional input of energy[3].

First, night-time adsorption and the processing of air can be managed through the natural flow of air encountered in open areas (wind). For instance, for the representative conditions for our experiments (30% RH and 25 °C) during night-time adsorption, the water content in air is approximately 0.006 kg of water per kg of air. Assuming the MOF layer is freely exposed to the natural flow of air at a calm wind speed of 0.3 m/s flowing onto the layer, the incident vapour flux at this condition is 0.003 kg m^{-2} s^{-1} (or 10.8 kg m^{-2} $hour^{-1}$). For the optimized MOF layer porosity (0.67) and thickness (~3 mm) from Supplementary Figure 7, the amount of MOF-801 is ~1.4 kg per m^2. The average flux of vapour adsorption shown in Supplementary Figure 7 is ~2E-5 kg m^{-2} s^{-1}. The approximately two orders of magnitude difference between the incident vapour and the vapour adsorption flux confirms that the natural flow of air is sufficient to ensure complete night-time adsorption.

During day-time operation, the dissipation of heat from the condenser to the ambient can also be managed by passive means of buoyant convection and the natural flow of air. Though, it is a common practice to assume ambient temperature condensers for thermodynamic analysis, we show a simple analysis to indicate that passive operation is possible. We start by developing an energy balance during the steady-state operation which can be expressed as

$$\dot{Q}_{\text{dissipation}} = \dot{Q}_{\text{condensation}} + \dot{Q}_{\text{gain}} \tag{12}$$

where $\dot{Q}_{\text{dissipation}}$ is the rate of heat dissipation from a finned heatsink to the ambient, $\dot{Q}_{\text{condensation}}$ is the rate of heat released during the condensation of water, and $\dot{Q}_{\text{gain}}$ is the rate of heat addition from the MOF layer to the condenser. Eqn (12) can be expressed as

$$h_{\text{dissipation}} A_{\text{heatsink}} (T_{\text{condenser}} - T_{\text{ambient}}) = \dot{m}_{\text{water}} h_{\text{fg}} + h_{\text{gain}} A_{\text{condenser}} (T_{\text{MOF}} - T_{\text{condenser}}) \tag{13}$$

In Eqn (13), h, A, and T are the heat transfer coefficient in W m^{-2} K^{-1}, heat transfer area in m^2, and temperature, respectively. $\dot{m}_{\text{water}}$ is the rate of condensation. Here, we assume an equal area of the MOF layer and condenser. In order to estimate the required heat transfer coefficient ($h_{\text{dissipation}}$) to enable passive operation, we assume a reasonable area ratio ($A_{\text{heatsink}}/A_{\text{condenser}}$) of 20,[11], and a temperature difference of 5 K between the condenser and the ambient. $\dot{m}_{water}$ is estimated based on complete desorption in 1 hour (~1E-4 kg m^{-2} s^{-1} or ~0.36 L m^{-2} $hour^{-1}$). Based on the experimentally measured temperatures shown in Figure 3b, for $T_{\text{MOF}} = 100$°C, $T_{\text{ambient}} = 35$°C and $h_{\text{gain}} = 10$ W m^{-2} K^{-1}, $T_{\text{condenser}}$ can be maintained at 40°C with an $h_{\text{dissipation}}$ of only ~10 W m^{-2} K^{-1}. This confirms that passive operation is achievable with buoyant convection and the naturally occurring flow of air.

Supplementary References

1 Narayanan, S., Yang, S., Kim, H. & Wang, E. N. Optimization of adsorption processes for climate control and thermal energy storage. *International Journal of Heat and Mass Transfer* **77**, 288-300 (2014).

2 Narayanan, S. *et al.* A thermophysical battery for storage-based climate control. *Applied Energy* **189**, 31-43 (2017).

3 Kim, H. *et al.* Water harvesting from air with metal-organic frameworks powered by natural sunlight. *Science* **356**, 430-434 (2017).

4 Welty, J. R., Wicks, C. E., Rorrer, G. & Wilson, R. E. *Fundamentals of momentum, heat, and mass transfer*. (John Wiley & Sons, 2009).

5 Moldrup, P. *et al.* Predicting the gas diffusion coefficient in repacked soil water-induced linear reduction model. *Soil Science Society of America Journal* **64**, 1588-1594 (2000).

6 Sircar, S. & Hufton, J. Why does the linear driving force model for adsorption kinetics work? *Adsorption* **6**, 137-147 (2000).

7 Kim, H. *et al.* Characterization of adsorption enthalpy of novel water-stable zeolites and metal-organic frameworks. *Scientific reports* **6** (2016).

8 Alonso, M., Sainz, E., Lopez, F. & Shinohara, K. Void-size probability distribution in random packings of equal-sized spheres. *Chemical engineering science* **50**, 1983-1988 (1995).

9 de Lange, M. F., Verouden, K. J., Vlugt, T. J., Gascon, J. & Kapteijn, F. Adsorption-Driven Heat Pumps: The Potential of Metal–Organic Frameworks. *Chemical reviews* **115**, 12205-12250 (2015).

10 Crank, J. *The mathematics of diffusion*. (Oxford university press, 1979).

11 Bar-Cohen, A., Iyengar, M. & Benjaafar, S. Design for manufacturability of natural convection cooled heat sinks. *International Journal of Transport Phenomena* **4**, 43-58 (2002).
