# Supplementary material for: Adsorption-based atmospheric water harvesting device for arid climates
Source: Nat Commun. 2018 Mar 22;9:1191. doi: 10.1038/s41467-018-03162-7 (PMC5864962; doi:10.1038/s41467-018-03162-7)
Supplement: Supplementary file 2 — Description of Additional Supplementary Files [file 41467_2018_3162_MOESM2_ESM.pdf]

Description of Additional Supplementary Files

File Name: Supplementary Movie 1
Description: Water harvesting cycle 2.

File Name: Supplementary Movie 2
Description: Water harvesting cycle 3.
